# Feature Selection in the Reconstruction of Complex Network Representations of Spectral Data

**DOI:** 10.1371/journal.pone.0072045

**Published:** 2013-08-26

**Authors:** Massimiliano Zanin, Ernestina Menasalvas, Stefano Boccaletti, Pedro Sousa

**Affiliations:** 1 Faculdade de Ciências e Tecnologia, Departamento de Engenharia Electrotécnica, Universidade Nova de Lisboa, Lisboa, Portugal; 2 Innaxis Foundation and Research Institute, Madrid, Spain; 3 Center for Biomedical Technology, Technical University of Madrid, Madrid, Spain; 4 CNR-Institute of Complex Systems, Florence, Italy; Tel Aviv University, Israel

## Abstract

Complex networks have been extensively used in the last decade to characterize and analyze complex systems, and they have been recently proposed as a novel instrument for the analysis of spectra extracted from biological samples. Yet, the high number of measurements composing spectra, and the consequent high computational cost, make a direct network analysis unfeasible. We here present a comparative analysis of three customary feature selection algorithms, including the binning of spectral data and the use of information theory metrics. Such algorithms are compared by assessing the score obtained in a classification task, where healthy subjects and people suffering from different types of cancers should be discriminated. Results indicate that a feature selection strategy based on Mutual Information outperforms the more classical data binning, while allowing a reduction of the dimensionality of the data set in two orders of magnitude.

## Introduction

The analysis of mass-spectrometrics data [1] is an old technique, dating back to 1958 [2], which is currently being used in a vast range of biomedical applications: from proteins [3] and metabolites [4] characterization, up to pharmacokinetics [5] and drug discovery [6]. Recently it has been proposed that the analysis of spectral data can be efficiently performed by means of *complex network* representations [7].

Networks [8], [9] are very simple mathematical objects, constituted by a set of nodes connected by links. Due to their simplicity and generality, they have become an invaluable tool for the analysis of complex systems, i.e., systems composed of a large number of elements interacting in a non-linear fashion, leading to the appearance of global *emergent behaviors* [10]. Applications range from the analysis of the dynamics of the human brain [11], social networks [12], up to transportation systems [13]. The interested reader may refer to several reviews that have been recently published on such topic, like for instance [14], [15].

As proposed in Ref. [7], spectral data can be transformed into networks, where nodes represent spectral measurements, and pairs of them are connected when their intensities follow a pattern associated with the disease under study. The structural analysis of the resulting network allows extracting relevant knowledge about the relationships between measurements characterizing the disease, and about their evolution through time. Yet, such direct representation comes at the cost of a high computational complexity: due to the high dimensionality of spectral data, which may include thousands of measurements for each subject, real-time processing is unfeasible. Furthermore, it is known that spectral data include a considerable quantity of noisy and irrelevant information, which make the analysis further challenging.

From the Knowledge Discovery field it is known that a high dimensionality of the feature space, like the case of large complex network representations, can make any learning problem more difficult. Indeed, even if many data mining algorithms attempt to automatically detect which features are important, and which features can be eliminated, both theoretical and experimental studies indicate that many algorithms scale poorly with a large number of irrelevant features are included [16]. The same problem is expected when analyzing network representations of spectral data: important features, e.g. specific topological characteristics, may be masked by the presence of nodes randomly connected, that is, codifying noise. The classical solution deals with the inclusion of a pre-processing step before the actual analysis of data: the *feature selection* phase [17]. The goal of the numerous techniques available in the Literature [18]–[20] is threefold: reducing the amount of data to be analyzed, center the analysis only on relevant data, and improve the quality of the data set. Feature selection has been especially useful in those domains that entail a large number of measured variables but a very low number of samples, like, for instance, biological and medical domains: gene and protein expressions, magnetoencephalographic and electroencephalographic records, and so forth.

The purpose of this study is to investigate the application of feature selection techniques in the reconstruction of complex network representations of spectral data. Three approaches commonly used in the Literature are investigated: from simple binning of the spectra, up to the application of information theory metrics. The effectiveness of such techniques is assessed by analyzing and comparing the score obtained in a classification task, which tries to discriminate control subjects from patients suffering from different types of cancer. Finally, the characteristics of the resulting networks are analyzed and discussed.

## Materials and Methods

### Cancer mass-spectrometrics data

The assessment of the effectiveness of the three feature selection algorithms has been performed against the *ARCENE* data set, as used in the NIPS 2003 feature selection challenge [21]. The training part of this data set included information for 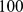 subjects, 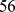 of them being control (healthy) subjects and 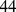 corresponding to people suffering from different kinds of cancers. Each one of them is described by a vector of 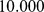 measurements, representing mass-spectra obtained with the SELDI technique [22].

Besides of the large number of measurements available for each subject, the challenge behind this data set resides in the presence of different types of cancers, i.e. ovarian and prostate cancers [23]–[25]. While its study may yield features that are generic of the separation cancer vs. control across various cancers, it also requires the classification method to take into account potential differences in disease, gender, and sample preparation.

### Feature selection

In this work, we propose the use of three different techniques for selecting a subset of the original 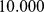 features that will be used in the classification process. The three techniques, as described in the remainder of this Section, have been selected due to their widespread use in spectra pre-processing and analysis. In addition, and in order to estimate the optimal network size required by each feature selection algorithm, four different network sizes have been considered: 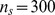, 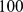, 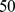 and 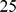 nodes.

The first feature selection technique here discussed is the *binning* of the data set, a technique widely used in the analysis of metabolic spectra [26], [27]. The original spectra were divided into sequential, non-overlapping regions; each one of these regions is converted into a new feature, whose value corresponds to the average of all measurements included in it.

The other two considered techniques are based on *Mutual Information* (MI for short), a well-known measure of mutual dependance between random variables [28], which has been extensively used for the selection of relevant features in a data set-see, for instance, Refs. [29]–[31]. Given two random variables 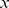 and 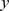, the two marginal probabilities distribution functions, 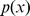 and 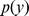, and the joint probability distribution function 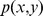, the mutual information 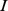 between 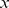 and 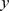 is defined as:
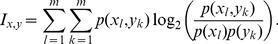
(1)

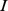
 measures, in bits, how much information is shared by two variables, i.e., how much the knowledge of one of them reduces the uncertainty about the other. In order to rank each feature included in the original data set, we create a metric assessing the average information shared by one feature with all the others:



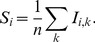
(2)At this point, there are two different possible approaches for selecting features based on their value of 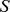. The first one, also known as the principle of *minimal redundancy* [31], states that the selected features should share the minimum amount of information between them, thus ensuring that the addition of a new feature provides new information to the classification process. This is equivalent to selecting features with small 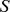, or to sorting them in an increasing order of 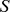. On the other hand, it is known that measurements obtained through mass spectrometry are characterized by a high degree of noise. When a measurement is representing noise, and thus no valuable information for the analysis, the quantity of information it shares with other measurements is expected to be small. Therefore, features with low 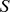 may codify no relevant information, while those associated with high 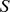 may form groups of highly correlated, and yet meaningful features.

Following these criteria, two different strategies are here compared for selecting features based on Mutual Information: select the 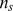 nodes with higher 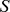, and the 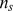 nodes with lower 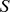.

### Network creation and characterization

The information available for each subject is here represented and analyzed by means of a complex network [8], [9], following the methodology recently proposed in Ref. [7]. A network is created for each subject, representing his/her healthy status; within this network, each node represents one of the selected measurements, as obtained by the three studied feature selection algorithms previously described, and links between pairs of nodes are created whenever the corresponding measurements exhibit characteristics found in patients. In what follow, such reconstruction technique is briefly described: the interested reader may refer to Ref. [7] for further details.

**Figure 1:**
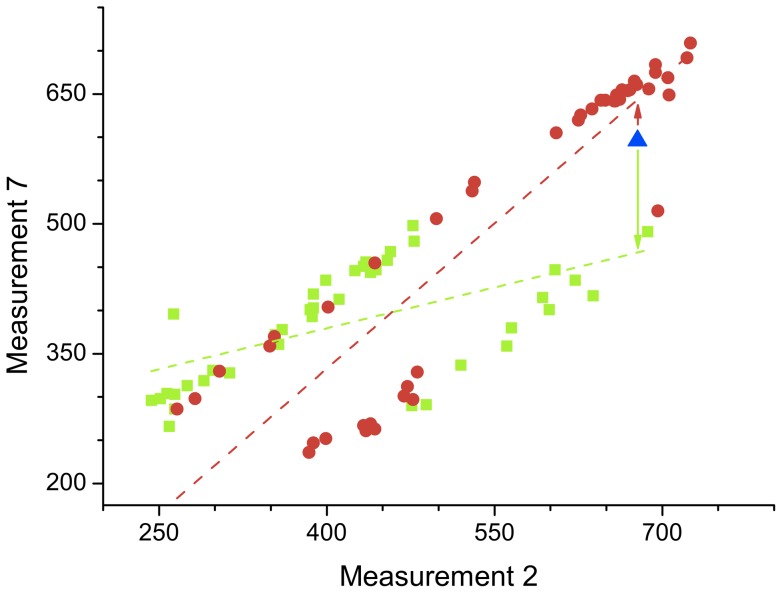
Reconstruction of the link weight between measurements 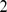 and 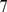. Green squares (red circles) represent the values of these two measurements for control and cancer subjects. The blue triangle corresponds to an unlabeled subject. Values correspond to the 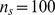 data set obtained through MI feature selection.

The methodology starts by associating a node to each one of the measurements available in the data set (or to each bin, in the case of data the first feature selection algorithm previously proposed). Links between pairs of nodes are created whenever the two corresponding measurements show a behavior consistent with a model extracted from cancer subjects, and sufficiently different from a model representing control subjects. These two models can be easily constructed by means of a linear correlation between pairs of measurements corresponding to control and cancer groups. Specifically, we linearly fit the values of the two measurements (in what follows, 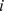 and 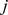) for both groups of labeled subjects:
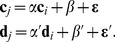
(3)

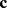
 and 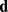 respectively represent data corresponding to control and cancer subjects, 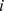 and 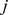 being the values of their 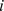-th and 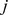-th measurements. Furthermore 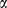 and 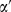 are the slopes of the two lineal fits (respectively, for the control and patient groups), 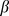 and 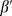 the two intercepts, and 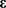 and 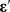 two vectors with the residuals of the fits. This step is represented in Fig. 1: green squares (red circles) represent the pairs of values under analysis for control subjects (subjects suffering from cancer), and green and red dashed lines the best lineal fit for each group. Notice that these lines represent the expected behavior of the two measurements under analysis in each group of data, i.e. the models that describe the expected relationship between pairs of measurement in control subjects and patients respectively. The problem of the creation of a link between these two measurements for a new subject is then equivalent to the identification of the model (line) to which his/her values are closer. The position of such unlabeled subject is indicated in Fig. 1 by the blue triangle. Two arrows, in red and green, represent the distance of this new subject from lineal fits corresponding to cancer and control subjects.

Taking into account the distance of the new subject from both models, and the corresponding expected error in the lineal fit (given by the standard deviation of residuals), the probability 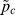 (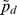) for the unlabeled subject of pertaining to the control (patient) group is proportional to the value of the corresponding normal distribution at the point defined by the second measurement (in Fig. 1, measurement 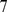). As the unlabeled subject must be classified into one of the two classes, the final probability of belonging to the patient class is given by the normalization:
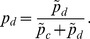
(4)

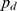
 therefore represents the likelihood for the relation between the two analyzed measurements of the unlabeled subject to belong to the model extracted from the cancer group. When this process is repeated for all 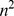 pairs of nodes, the result is a weighted clique, i.e. a weighted fully-connected network, representing the subject under study.

**Figure 2:**
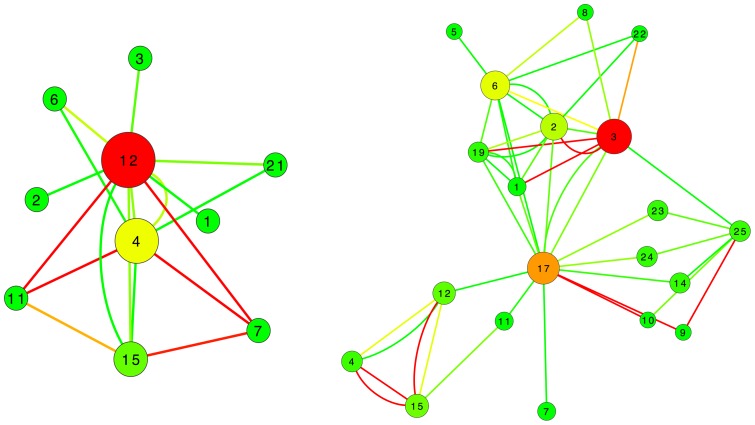
Example of reconstructed networks. Left (Right) graph depicts the network corresponding to a control subject (patient), for 25 bins selected in decreasing Mutual Information order. Nodes color and size represent their degree, while link color codifies the corresponding strength. For sake of clarity, only links with strength greater than 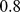 are represented.

Fig. 2 reports the graphical representation of two networks created by means of the described methodology, corresponding to a control subject (Left) and a patient (Right), after the most important 25 features have been selected by means of a decreasing Mutual Information feature selection schema. For the sake of clarity, only links with strength greater than 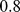 are represented. These two examples already show important differences in the structures characterizing control subjects and patients, e.g. the higher number of links present in the patient network.

In order to analyze in a more systematical way this resulting clique, we apply the method proposed in Ref. [32], which involves two steps: (*i*) apply different thresholds to the weighted clique, in order to obtain different unweighted networks of different link densities, and (*ii*) extract a large set of topological metrics from each one of them. Such topological metrics are then fed inside different classification algorithms, with the aim of distinguish between healthy and cancer subjects.

## Results and Discussion

**Figure 3:**
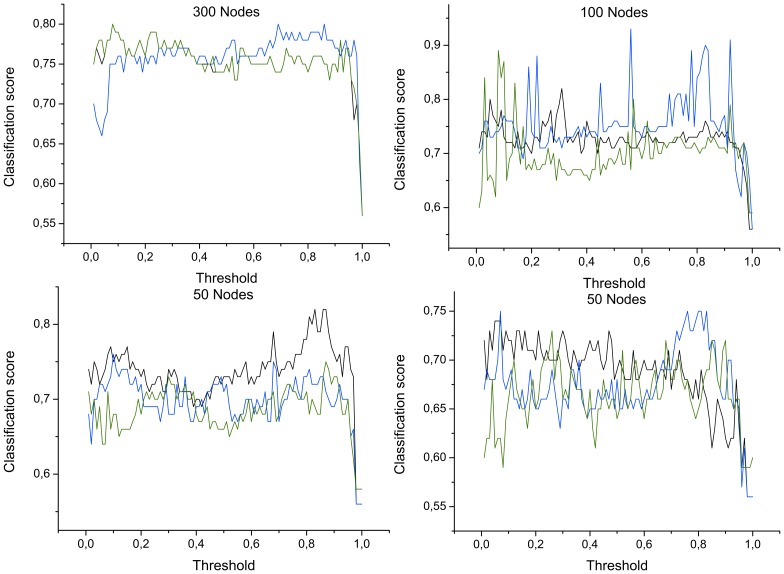
Classification scores as a function of the number of nodes and of the applied threshold. Black, blue and green lines respectively represent the classification score (*precision*) obtained by averaged bins, and by measurements selected in decreasing and increasing Mutual Information order.

**Figure 4:**
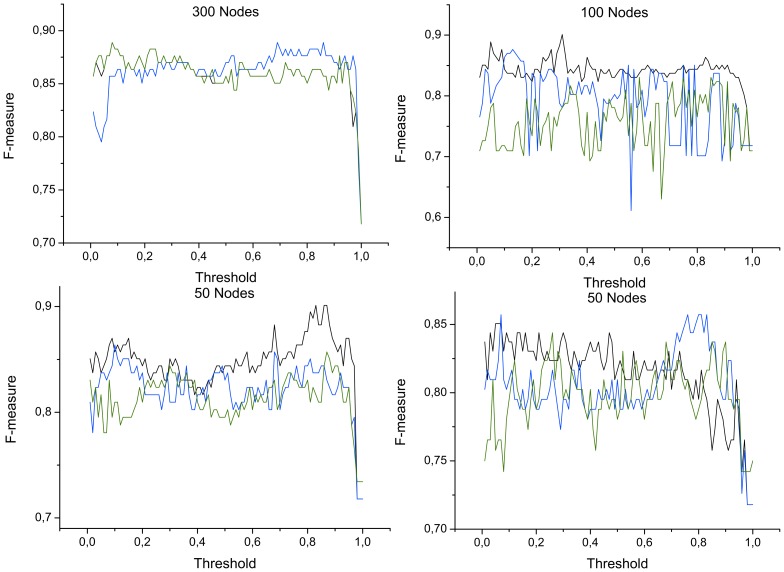
F-measure as a function of the number of nodes and of the applied threshold. Black, blue and green lines respectively represent the classification score (*precision*) obtained by averaged bins, and by measurements selected in decreasing and increasing Mutual Information order. See main text for the definition of the *F-measure*.

Fig. 3 shows the classification score, expressed by means of the *precision* of the classification, as a function of the applied threshold and in the different scenarios here analyzed. Specifically, each image composing Fig. 3 reports the results corresponding to the four network sizes here considered: from left to right, top to bottom, 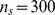, 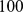, 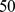 and 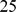. Furthermore, inside each graph the three lines represent the score associated to the network representation created by means of the three feature selection algorithms here considered: average binning, measurements with high 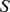, and measurements with low 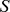. In this case, the selection has been performed by means of a *Support Vector Machine* algorithm [33], due to its simplicity and its effectiveness in identifying relevant network metrics [32]. Fig. 4 reports the quality of the classification expressed in terms of the *F-measure* [34], defined as:
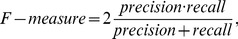
(5)
*recall* being the number of correct results divided by the number of results that should have been returned. While some minor differences can be detected, especially in the behavior of the classification with 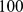 nodes, a general agreement between Figs. 3 and 4 is observed.

**Figure 5:**
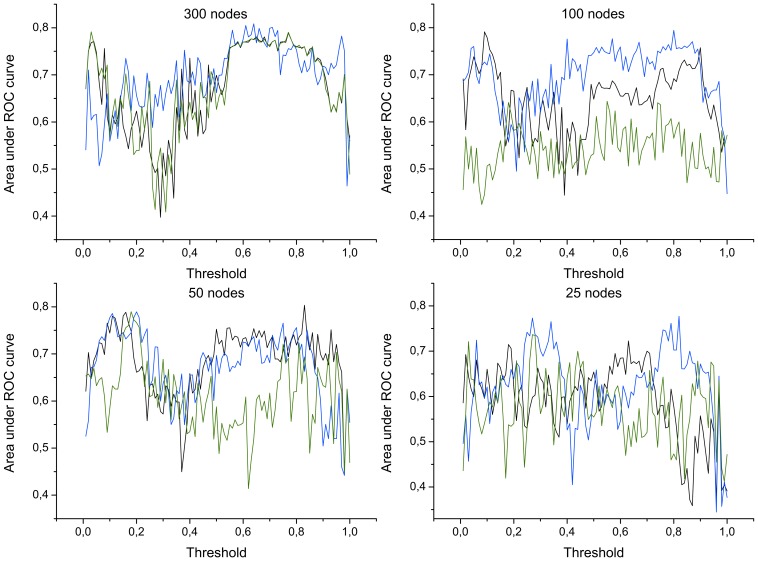
Area under the ROC curve, as a function of the number of nodes and of the applied threshold. Black, blue and green lines respectively represent the classification score (*precision*) obtained by averaged bins, and by measurements selected in decreasing and increasing Mutual Information order.

In order to validate such results, and exclude their dependence on the chosen classification algorithm, Fig. 5 represents the classification score obtained by means of *Probabilistic Neural Networks* [35], [36]. In this case, the result is given as the area under the ROC curve [37], which allows analyzing the performance of binary classifier systems whose output is expressed as a probability.

Finally, Table 1 reports a resume of the results, i.e. of the best classification score obtained as a function of the number of features included in the analysis (in this case, the number of nodes constituting the networks), and the feature selection algorithm applied. Several conclusions can be drawn from these results.

**Table 1:** Resume of the best classification scores.

	Average	Mutual Information (high *S*)	Mutual Information (low *S*)
**300 nodes**	0.8	0.8	0.8
**100 nodes**	0.82	0.93	0.89
**50 nodes**	0.82	0.76	0.75
**25 nodes**	0.74	0.75	0.73

First of all, reducing the number of measurements included in the analysis improves the classification score. Clearly, reducing the dimensionality of the data set under analysis allows limiting the quantity of noise, i.e. irrelevant information, included in it, thus simplifying the classification task. Furthermore, reducing the number of features beyond a given threshold results in a drop in the effectiveness of the classification; this also has to be expected, in that important information for the task may be deleted. Such threshold is higher in the case of MI-based feature selection algorithms, which display their maximum for networks of 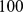 nodes.

MI-based feature selection algorithms are more effective than a feature reduction based on binning, as shown by the higher classification scores (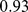 vs. 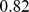). This indicates that creating bins by averaging the measurements inside sequential regions, while a common practice in the study of biological spectra, may result in the deletion of important information, which can be codified in very small windows or even in single measurements. While MI-based feature selection strategies always yield better results, the best solutions are obtained by selecting measurements with higher 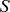. Therefore, the important information is codified within few measurements that are highly correlated between them; on the contrary, selecting measurements according to a *minimal redundancy* strategy seems to introduce a high amount of noise in the classification task, reducing the discrimination power.

Besides its intrinsic value, the score obtained in the classification task also provides information about the best network representation: such information, in turns, can be used to understand which are the relationships between measurements that characterize the disease under study. A closer look at Figs. 3 and 5 suggests that there is a common pattern in the evolution of classification scores and areas under the ROC curve. Specifically, two local maxima are usually present, one corresponding to low thresholds (between 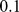 and 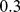), and a second one to high threshold values (between 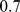 and 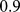). This pattern is consistent across different network sizes, and is especially relevant in the case of networks whose nodes are measurements with high 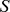. In this case, the correlation between the score curve (Fig. 3) and the corresponding area under the ROC curve (Fig. 5) lies within the interval 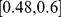, while the correlation between the scores obtained by different network sizes lies between 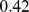 and 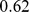. Such maxima are associated to different network topologies, and to different topological metrics. Specifically, when a low threshold is applied, most links are present in the network, creating a dense graph; in such cases, it is possible to analyze local structures, like for instance *motifs* [38], thus providing information about the micro-scale interactions between a small number of measurements. On the other hand, when a high threshold is applied, the resulting networks have a sparse structure, which does not allow the identification of micro-structures; on the contrary, most information is codified in the macroscopic structure of the networks, with metrics like *diameter of the network* [8], [9] of their *modular structure* [39] being the most important.

## Conclusions

The present study compares the application of three different feature selection algorithms to the analysis of mass-spectrometrics data by means of complex networks. Due to the high dimensionality of the initial data set, comprising 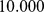 different measurements for each subject, a direct network representation of such data would be unfeasible, by reason of the extremely high computational cost associated to the analysis of graphs with thousands of nodes. Furthermore, it is known that spectral data contain a high quantity of redundant and noisy information, which can be safely eliminated, and whose presence may even reduce the discrimination capability of a classification algorithm. While commonly used in the Literature, our results indicate that binning the spectrum, i.e. considering the average of sequential non-overlapping regions, yields to a destruction of relevant information. On the contrary, a MI-based feature selection algorithm can be used to safely reduce the number of measurements, and therefore of nodes in the network representation, in two orders of magnitude.

## References

[1] Hoffmann E (1996) Mass spectrometry. Wiley Online Library.

[2] Andersson CO (1958) Mass spectrometric studies on amino acid and peptide derivatives. Acta chem scand 12.

[3] Link AJ, Eng J, Schieltz DM, Carmack E, Mize GJ, et al (1999) Direct analysis of protein complexes using mass spectrometry. Nature biotechnology 17: 676–682. 10.1038/10890 10404161

[4] Dettmer K, Aronov PA, Hammock BD (2007) Mass spectrometry-based metabolomics. Mass spectrometry reviews 26: 51–78. 16921475 10.1002/mas.20108 PMC1904337

[5] Covey TR, Lee ED, Henion JD (1986) High-speed liquid chromatography/tandem mass spectrometry for the determination of drugs in biological samples. Analytical Chemistry 58: 2453–2460. 3789400 10.1021/ac00125a022

[6] Moseley III MA, Sheeley DM, Blackburn RK, Johnson RL, Merrill BM (1998) Mass spectrometry in drug discovery. Mass Spectrometry of Biological Materials 2: 162.

[7] Zanin M, Papo D, Solís JLG, Espinosa JCM, Frausto-Reyes C, et al (2013) Knowledge discovery in spectral data by means of complex networks. Metabolites 3: 155–167. 24957895 10.3390/metabo3010155 PMC3901251

[8] Newman ME (2003) The structure and function of complex networks. SIAM review 45: 167–256.

[9] Boccaletti S, Latora V, Moreno Y, Chavez M, Hwang DU (2006) Complex networks: Structure and dynamics. Physics reports 424: 175–308.

[10] Anderson PW (1972) More is different. Science 177: 393–396. 17796623 10.1126/science.177.4047.393

[11] Bullmore E, Sporns O (2009) Complex brain networks: graph theoretical analysis of structural and functional systems. Nature Reviews Neuroscience 10: 186–198. 19190637 10.1038/nrn2575

[12] Scott J (2012) Social network analysis. SAGE Publications Limited.

[13] Zanin M, Lillo F (2013) Modelling the air transport with complex networks: A short review. The European Physical Journal Special Topics 215: 5–21.

[14] Costa LdF, Oliveira Jr ON, Travieso G, Rodrigues FA, Villas Boas PR, et al (2011) Analyzing and modeling real-world phenomena with complex networks: a survey of applications. Advances in Physics 60: 329–412.

[15] Havlin S, Kenett D, Ben-Jacob E, Bunde A, Cohen R, et al (2012) Challenges in network science: Applications to infrastructures, climate, social systems and economics. The European Physical Journal Special Topics 214: 273–293.

[16] Langley P (1996) Elements of machine learning. Morgan Kaufmann.

[17] Guyon I, Elisseeff A (2003) An introduction to variable and feature selection. The Journal of Machine Learning Research 3: 1157–1182.

[18] Kira K, Rendell LA (1992) The feature selection problem: Traditional methods and a new algorithm. In: Proceedings of the National Conference on Artificial Intelligence. John Wiley & Sons Ltd, 129–129.

[19] Saeys Y, Inza I, Larrañaga P (2007) A review of feature selection techniques in bioinformatics. Bioinformatics 23: 2507–2517. 17720704 10.1093/bioinformatics/btm344

[20] Liu H, Motoda H (2007) Computational methods of feature selection. Chapman and Hall/CRC.

[21] Guyon I, Gunn S, Ben-Hur A, Dror G (2004) Result analysis of the nips 2003 feature selection challenge. Advances in Neural Information Processing Systems 17: 545–552.

[22] Issaq HJ, Veenstra TD, Conrads TP, Felschow D (2002) The seldi-tof ms approach to proteomics: protein profiling and biomarker identification. Biochemical and biophysical research communications 292: 587–592. 11922607 10.1006/bbrc.2002.6678

[23] Petricoin III EF, Ardekani AM, Hitt BA, Levine PJ, Fusaro VA, et al (2002) Use of proteomic patterns in serum to identify ovarian cancer. The lancet 359: 572–577. 10.1016/S0140-6736(02)07746-2 11867112

[24] Petricoin EF, Ornstein DK, Paweletz CP, Ardekani A, Hackett PS, et al (2002) Serum proteomic patterns for detection of prostate cancer. Journal of the National Cancer Institute 94: 1576–1578. 12381711 10.1093/jnci/94.20.1576

[25] Adam BL, Qu Y, Davis JW, Ward MD, Clements MA, et al (2002) Serum protein fingerprinting coupled with a pattern-matching algorithm distinguishes prostate cancer from benign prostate hyperplasia and healthy men. Cancer Research 62: 3609–3614. 12097261

[26] Griffin J, Williams H, Sang E, Clarke K, Rae C, et al (2001) Metabolic profiling of genetic disorders: A multitissue (1) H nuclear magnetic resonance spectroscopic and pattern recognition study into dystrophic tissue. Analytical Biochemistry 293: 16–21. 11373073 10.1006/abio.2001.5096

[27] Beckonert O, E Bollard M, Ebbels T, Keun HC, Antti H, et al (2003) Nmr-based metabonomic toxicity classification: hierarchical cluster analysis and k-nearest-neighbour approaches. Analytica Chimica Acta 490: 3–15.

[28] Karmeshu J (2003) Entropy measures, maximum entropy principle and emerging applications. Springer-Verlag New York, Inc.

[29] Yang Y, Pedersen JO (1997) A comparative study on feature selection in text categorization. In: Machine Learning-International Workshop Then Conference. Morgan Kaufmann Publishers, Inc., 412–420.

[30] Fleuret F (2004) Fast binary feature selection with conditional mutual information. The Journal of Machine Learning Research 5: 1531–1555.

[31] Peng H, Long F, Ding C (2005) Feature selection based on mutual information criteria of maxdependency, max-relevance, and min-redundancy. Pattern Analysis and Machine Intelligence, IEEE Transactions on 27: 1226–1238. 10.1109/TPAMI.2005.159 16119262

[32] Zanin M, Sousa P, Papo D, Bajo R, García-Prieto J, et al.. (2012) Optimizing functional network representation of multivariate time series. Scientific reports 2. 10.1038/srep00630 PMC3433690 22953051

[33] Hamel LH (2011) Knowledge discovery with support vector machines, volume 3. Wiley-Interscience.

[34] Powers D (2011) Evaluation: From precision, recall and f-measure to roc., informedness, markedness & correlation. Journal of Machine Learning Technologies 2: 37–63.

[35] Clark JW (1988) Probabilistic neural networks. Evolution, Learning and Cognition: 129–180.

[36] Specht DF (1990) Probabilistic neural networks. Neural networks 3: 109–118. 10.1109/72.80210 18282828

[37] Zweig MH, Campbell G (1993) Receiver-operating characteristic (roc) plots: a fundamental evaluation tool in clinical medicine. Clinical chemistry 39: 561–577. 8472349

[38] Milo R, Shen-Orr S, Itzkovitz S, Kashtan N, Chklovskii D, et al (2002) Network motifs: simple building blocks of complex networks. Science Signaling 298: 824. 10.1126/science.298.5594.824 12399590

[39] Fortunato S (2010) Community detection in graphs. Physics Reports 486: 75–174.

